# Autofluorescence in posterior uveitis

**DOI:** 10.4103/0301-4738.67040

**Published:** 2010

**Authors:** Jay Kumar Chhablani

**Affiliations:** L V Prasad Eye Institute, Hyderabad, India

Dear Editor,

I read the article by Sudharshan *et al*.,[1] which describes the current approach in posterior uveitis. I would like to congratulate the authors for an excellent comprehensive review and to add a few comments about autofluorescence (AF) in various posterior uveitic conditions.

The amount of AF is determined by the amount of fluorophores, which varies during the acute and resolution phases of inflammation. Hypertrophy and reactive hyperplasia of retinal pigment epithelium (RPE) is associated with increase in AF, due to accumulation of flurophores. A transmission defect during fundus fluorescein angiography (FFA), because of RPE atrophy, appears to be hypo-autofluorescencent.

Following are the important AF findings reported in various chorioretinal inflammatory conditions:

1. **Acute posterior multifocal placoid pigment epitheliopathy (APMPPE):** In the acute phases of APMPPE, AF imaging shows more lesions as compared to FFA or ophthalmoscopy. This indicates that RPE damage occurs secondary to choroidal changes. During the followup, many lesions developed increased pigmentation centrally, with a depigmented halo clinically. The central portions of the lesions appeared to have increased pigment and showed hyper-autofluorescence. The depigmented halo appeared to show decreased, almost absent AF, suggesting atrophy or absence of functional RPE cells.
2. **Multiple evanescent white dot syndrome (MEWDS):** In the acute phase of MEWDS, AF photography shows less numerous, but increased AF areas, corresponding to the focal hypocyanescent spots seen on indocyanine green angiography (ICG), probably due to the excitation of the photoreceptor-retinal pigment epithelium complex. Following resolution of the lesions, AF and ICG return to a normal pattern.[2]
3. **Acute syphilitic posterior placoid chorioretinitis:** A yellow-white placoid lesion in the macular area in this disease shows increased AF, which may be due to the accumulation of lipofuscin in the RPE or to imperfect phagocytosis and processing of the photoreceptor outer segments in the acute phases of the disease. With treatment, the yellow color, opacification of the retina, increases the autofluorescence resolve.[3]
4. **Multifocal choroiditis and panuveitis (MCP):** In MCP, AF shows numerous hypo-autofluorescent spots corresponding to the clinically visible chorioretinal scars, but more in number.[4] This indicates more extensive RPE damage compared to the clinically visible damage.
5. **Serpiginous choroiditis (SC):** In SC, hyper-autofluorescence is seen two to five days after the appearance of the lesions, which progressively decreases during the scarring phase of the disease.
6. **Acute zonal occult outer retinopathy (AZOOR):** Zonal damage in AZOOR is characterized by an intensely autofluorescent outer border of the affected zone, consistent with the presence of lipofuscin and a hypo-autofluorescent central area, consistent with atrophy of the RPE.[5]
7. **Birdshot chorioretinopathy:** In this disease the early lesions do not necessarily cause autofluorescent abnormalities, but hypo autofluorescence is usually associated with the depigmented lesions suggesting RPE atrophy. Linear hypo autofluorescent streaks along the retinal vessels and placoid hypo autofluorescence in the macula can also be seen.
8. **Vogt-Koyanagi-Harada disease:** Serous detached areas show hypo-autofluorescence due to the blockage. After resolution of the serous detachment, numerous hypo-autofluorescence granular dots correspond to the window defects on FFA and areas of RPE damage or atrophy, clinically.
